# Medicinal Plants Used for Combating Human Bacterial Diseases in Ethiopia: A Systematic Review

**DOI:** 10.1155/tswj/1278796

**Published:** 2026-08-28

**Authors:** Derebe Alemneh

**Affiliations:** ^1^ Department of Biology, Injibara University, Injibara, Ethiopia, inu.edu.et

**Keywords:** antibacterial, antidotes, bacterial diseases, medicinal plants, phytochemicals

## Abstract

In Ethiopia, bacterial diseases cause the majority of illness and mortality. They have become great challenges to the country, and a great proportion of its population are utilizing medicinal plants as sources of medicine for fighting diseases. The aim of the present work was to present a compiled document to show the great potential of plants in fighting bacterial diseases for promoting future exploration, phytochemical and antibacterial test studies. To prepare the work, three steps were designed. First, ethnobotanical articles that were published starting from 2000 to 2025 were gathered from reputable databases: Google Scholar, PubMed, and AJOL. In the second step, screening and exclusion of the gathered articles were conducted. In the last step, data extraction and analysis were conducted. In this study, 168 medicinal plants (73 families) that were used to treat 10 bacterial diseases were recorded. Euphorbiaceae was the foremost family in species number, whereas *Calpurnia aurea* was the first as cited by 10 research works. The highest proportion of medicinal plants (89 species) were reported to treat diarrhea, followed by those used to treat gonorrhea (43 species). Leaf (38.2%) took the prime position as sources of traditional remedies. Many traditional remedies need several antidotes such as coffee, local alcohol, different parts and forms of plant species such as *Allium sativum*, *Lepidium sativum, Cucumis ficifolius,* and *Ensete ventricosum*. Medicinal plants were also reported with huge potential to be used as bactericidal chemicals. In conclusion, bacterial diseases are the greatest threat to Ethiopia and medicinal plants are the future hope for fighting them. Thus, further ethnobotanical exploration, phytochemical and antibacterial test studies should be conducted particularly on *Ajuga integrifolia, Gomphocarpus purpurascens, Tephrosia elata, C. ficifolius, Rhus vulgaris,* and *Rytigynia neglecta* used for the treatment of pneumonia and lung TB, respectively, for new drug discovery and sustainable utilization of the species.

## 1. Introduction

Different experimental studies conducted in Ethiopia showed its great number of the population is still suffering from bacterial diseases. According to [1], bacterial infections caused about 95.8% of all neonatal illnesses from 2018 to 2019. *Helicobacter pylori*, which is the main cause of gastrointestinal diseases, is the most prevalent in the country [2]. Tuberculosis is a major communal health concern in the country [3]. Various types of bacteria, including *Staphylococcus aureus*, *Haemophilus influenzae*, *Streptococcus pneumoniae*, and *Pseudomonas aeruginosa* cause bacterial eye infections in the country [4]. *Shigella* species, the causative agent of dysentery, has become the major cause of death in the country [5]. Other reports showed that antimicrobial‐resistant, carbapenem‐resistant *P*. *aeruginosa* is becoming a great threat to public health [6]. Nowadays, such bacterial diseases bring a great challenge to the country’s population due to their ability to resist different types of antibiotics, as discussed in different literature sources [7–10] beyond becoming an economic burden to the country [11].

Several ethnobotanical findings showed that 80% of the country′s population is continuing the utilization of traditional remedies derived from plants in treating bacterial diseases [12–14]. This is due to the presence of cultural acceptability of healers, the relatively low cost of traditional medicine, and difficult access to modern health facilities, especially in remote areas with low infrastructure access [15]. The research reports of [16–20] are among a lot of research findings that assure the application of huge numbers of medicinal plants, which are becoming sources of traditional medicine, for fighting different bacterial diseases in different parts of the country. Thus, there are such compiled and noncompiled reports glorifying the medicinal plants′ role in treating severe bacterial diseases. In recent days, various experimental work has been conducted on medicinal plants in the search of their phytochemicals, and for testing the antibacterial potential of the medicinal herbs, and the experimental work came up with good and hopeful results. These analytical studies show that the presence of huge types of phytochemicals in medicinal plants as observed in the works of [21–24]. There are also antimicrobial test studies that show the ability of medicinal plants to fight diseases, as discussed in some research findings of [25, 26].

However, there are no sufficient review works showing the compiled ethnobotanical, phytochemical, and antimicrobial test studies. Therefore, the objective of the current review focuses on presenting a compiled document by extracting different essential information from different ethnobotanical research findings, antimicrobial and phytochemical test studies. So, this work tried to fill the gap in showing the power of the traditional knowledge and medicinal plants in fighting bacterial diseases in the country, and their potential for antibacterial activity and to be sources of varied phytochemicals. The work is hopeful in promoting future phytochemical analysis and antimicrobial test studies resulting in future new pharmaceutical research.

## 2. Methodology

### 2.1. Data Gathering

Scientific articles with an ethnobotanical approach that were published from 2000 to 2025 and presented a list of medicinal plants used for the treatment of bacterial diseases in Ethiopia were gathered. The aim was to incorporate the 25 ethnobotanical data. An inclusive review was performed using reputable databases, Google Scholar, PubMed, and AJOL. The search keywords (search words) were “Medicinal plants and bacterial diseases in Ethiopia,” “Ethnobotanical study of medicinal plants in Ethiopia,” “Traditional medicinal plants and bacterial diseases in Ethiopia,” “Traditional medicine and bacterial diseases in Ethiopia,” “Phytochemical analysis of medicinal plants in Ethiopia,” and “Medicinal plants and antibacterial test in Ethiopia.”

### 2.2. Screening and Exclusion Criteria

The screening of different data sources was conducted step by step. (1) Articles found more than once in the search engine were excluded. (2) Unaccessible articles were excluded. (3) Articles without an ethnobotanical approach and done out of Ethiopia were excluded. (4) Review and unpublished articles were excluded. (5) Articles that did not present the scientific names of species were excluded. (6) Articles that did not match the present article in title were excluded.

(7) The screening steps listed from 1 to 6 were also applied for the screening of the 30 phytochemical and antibacterial test studies. In addition, research works with duplicated ideas, unclear data and incomplete information were excluded for antibacterial and phytochemical test studies.

### 2.3. Data Extraction and Analysis

The ethnobotanical data were extracted from 53 ethnobotanical findings. The author also utilized 30 additional research works for the analysis of different phytochemical (10 research works; two common) and antibacterial test studies (22 research works; two were common). Scientific, family and local name, parts used, disease treated, preparation and application method, and route of administration were extracted. The studied plants with the reserved phytochemicals, antibacterial activity, parts used, and the type of bacteria tested were also extracted from these studies. The plant′s scientific name was cross‐checked with the help of the Flora of Ethiopia and Eritrea (Volumes 1–8) and POWO (Plants of the World Online). The gathered data was analyzed using different analysis methods through Microsoft Excel 2010 spreadsheet software. The results were presented through tables, plots, and graphs.

## 3. The Findings of the Review

### 3.1. Location of the Study Areas in Ethiopia

The result of the present study showed that most of the findings (35.85%) incorporated as data sources for the current work were conducted in the Amhara region, followed by those findings (24.5%) done in the Oromia region and southern region (16.98%). A fewer number of studies from Somalia and Harar regions located in the eastern part of the country were also used as a data source for the study. As a result of the area coverage of the regions, there was variation in species records among the regions. This showed that most research work was conducted in the central part of the country, whereas least studies were conducted in its peripheral parts. This might be due to several factors, including the remoteness of the areas, especially from the previously established Ethiopian universities, security problems and the exclusion criteria. It further showed that diarrhea was the most distributed, hence the most prevalent bacterial disease in most parts of the study sites. This might be because this disease is strongly related to hygiene, which is difficult for the rural population living with limited access to water (Table 1).

**Table 1:** Reported diseases and frequency in each region.

Disease type	Region and disease frequency							
	AFR	AMR	BGR	HAR	OR	SNNPR	SR	TR
Chancroid	—	3	—	—	3	—	—	—
Diarrhea	2	57	NR	1	26	24	1	8
Dysentery	2	11	—	—	—	2	—	—
Gonorrhea	NR	5	3	NR	27	17	—	—
Leprosy	3	2	2	—	—	1	—	—
Pneumonia	—	2	—	—	5	4	—	—
Syphilis	NR	5	—	—	1	3	—	—
TB ^∗^	3	7	—	—	8	1	—	6
Typhoid	5	2	—	1	1	1	—	—

Abbreviations: —, not reported; AFR, Afar region; AMR, Amhara region; BGR, Benishangul region; HAR, Harar region; OR, Oromia region; SNNPR, Southern region; SR, Somalia region; TB ^∗^, include bone TB, lung TB, gland TB; TR, Tigray region.

### 3.2. Diversity of Medicinal Plants

#### 3.2.1. Species Diversity

A total of 168 traditional medicinal plant species grouped under 73 families were recorded from the 53 research findings used to traditionally treat 10 types of bacterial diseases in different parts of Ethiopia. More than half of the total families (57.5%) were recorded with single species (Table 2). This might be related to the number of species and distribution range in the flora area of Ethiopia.

**Table 2:** Family list with species number and percent.

S.No	Family name	NS	%	TF	TF%
1.	1. Acanthaceae 2.Amaryllidaceae 3.Araceae 4.Aristolochiaceae 5.Asclepiadaceae 6.Asparagaceae 7.Balanitaceae 8.Bignoniaceae 9. Brassicaceae 10. Burseraceae 11. Caricaceae 12. Celastraceae 13. Crassulaceae 14. Cupressaceae 15. Ebenaceae 16. Ericaceae 17. Francoaceae 18. Iridaceae 19. Linaceae 20. Loranthaceae 21. Myrsinaceae 22. Musaceae 23. Nyctaginaceae 24. Olacaceae 25. Oleaceae 26. Papaveraceae 27. Penaeaceae 28. Peraceae 29. Phytolaccaceae 30. Plumbaginaceae 31. Podocarpaceae 32. Polygalaceae 33. Primulaceae 34. Proteaceae 35. Ranunculaceae 36. Salvadoraceae 37. Santalaceae 38. Sapindaceae 39. Simaroubaceae 40. Tamaricaceae 41. Thymelaeaceae 42. Urticaceae 43. Zingiberaceae	1	0.6	43	58
2.	1. Amaranthaceae 2. Apiaceae 3. Asphodelaceae 4. Balsaminaceae 5. Capparaceae 6. Combretaceae 7. Menispermaceae 8. Polygonaceae 9. Rosaceae 10. Rutaceae 11. Scrophulariaceae 12. Vitaceae 13. Verbenaceae	2	1.2	13	17.8
3.	1. Anacardiaceae 2. Apocynaceae 3. Meliaceae 4. Moraceae 5. Poaceae 6. Rhamnaceae	3	1.8	6	8.2
4.	1. Rubiaceae 2. Boraginaceae 3. Myrtaceae	5	3	3	4.1
5.	1. Solanaceae 2. Cucurbitaceae	6	3.6	2	2.7
6.	1. Malvaceae	7	4.2	1	1.4
7.	1. Asteraceae	10	6	1	1.4
8.	1. Lamiaceae 2. Euphorbiaceae 3. Euphorbiaeae	11	6.6	3	4.1
9.	1. Fabaceae	12	7.2	1	1.4

Abbreviations: NS, number of species; TF, total family.

#### 3.2.2. Top Families in Species Number

**Figure 1:**
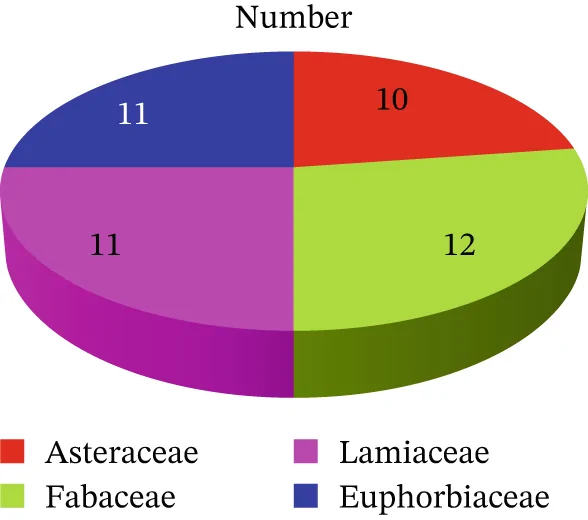
The first four leading families in species number.

The result showed that four families were recorded relatively with higher number of species than the remaining 69 families. Fabaceae was the dominant family followed by Euphorbiaceae, Lamiaceae, and Asteraceae (Figure 1). One of the reasons for Euphorbiaceae to be one of the leading families in species number might be because the species belonging to the family possess different essential phytochemicals, such as the diterpenoids, the triterpenoids, sterols, flavonoids, phenols and tannins, glycerides, flavonoids, phenols [27], alkaloids, cyanogenic glycosides, diterpenes, glucosinolates, tannins, and triterpenes [28], which increase the curative ability of the species to be selected as medicinal herb by the healers.

Taxonomically, Asteraceae is the first family, whereas Fabaceae is the third in species number in the world flora of angiosperms. This might make families able to catch and cover large areas of the world and to be available in most areas of the world, which is the same as true in Ethiopia. According to [29], Fabaceae has a cosmopolitan distribution species in the tropics, to which Ethiopia belongs. This distribution pattern makes the families more available to the community as medicinal herbs than other families with limited distributional range. In addition, the species belonging to Fabaceae, like the species belonging to Euphorbiaceae, contain different phytochemicals such as isoflavones, prenylated flavonoids, flavanones, flavanols, saponins, glucosides, rotenoids, chalcones, alkaloids, and trypsin inhibitors, even if flavonoids are the main constituents of the Fabaceae family. Because of this reason, species in the family have estrogenic, antibacterial, antioxidant, antifungal, and insecticidal activities [30].

Asteraceae is the most diverse family of angiosperms, having a cosmopolitan distribution [31]. Thus, it might be available to the community to be used as a source of traditional medicine. The other point is because of possessing essential bioactive chemical compounds such as polyphenols, flavonoids, terpenoids, and sesquiterpene lactones [32]. The volatile phytochemicals such as monoterpenoids such as camphor, *α*‐pinene, camphene, and 1, 8‐cineole and nonvolatile phytochemicals such as phenolcarboxylic and cinnamic acids and their derivatives, flavonoids (among which are flavones, isoflavones, flavanols, flavanones, flavanones, flavans, flavans 3,4‐diols, catechins, biflavonoids), and proanthocyanidins, glycosides, triterpenes, lignins, and aldehydes are found in Lamiaceae [33].

#### 3.2.3. Highly Cited Species

The result of the analysis showed that as the use proportion of the parts of medicinal plants varies across species (Table 3), the citation of species is also varied among the species. For instance, *Calpurnia aurea*, which belongs to Fabaceae, was the first species in citation number (Table 4). This indicates that the species is the most well‐known species in the country than the other recorded species. The results of the phytochemical analysis studies showed that the species was the source of various essential primary and secondary plant metabolites such as glycosides, carbohydrates, phenols, tannins, flavonoids, oils, fats, proteins, terpenoids, saponins, steroids, and alkaloids [92]. This might be one reason for the advanced distribution of the species in most parts of the country than the other recorded species. In other findings, the species was recorded to have good antibacterial activity [93].

**Table 3:** Parts, number, and percent of medicinal plants and disease types and causative agents.

No	Types of diseases	Causative agent	Citation	Number, parts used, and percent of the species										
				N0	%	F	L	R	Rh	St	Sd	Wp	Bu	La
1.	Chancroid	*Haemophilus ducreyi.*	[34]	2	1.7	—	—	100	—	—	—	—	—	—
2.	Diarrhea	*Escherichia coli*.	[35]	88	52	5	40	22	1	18	12.5	1.4	0	0
3.	Dysentery	*Shigella bacterium*.	[35]	12	7	7	43	29	—	7.1	7.1	—	—	7.1
4.	Gonorrhea	*Neisseria gonorrhoeae*.	[36]	41	25	2	34	48		10.7	1.8	—		3.6
5.	Leprosy	*Mycobacterium leprae*.	[37]	11	6	—	54	38	—	7.7	—	—	—	—
6.	Pneumonia	*Streptococcus pneumoniae*.	[38]	12	7	—	20	26.7	—	13.3	33.3	6.7	—	—
7.	Syphilis	*Treponema pallidum* subspecies *pallidum*.	[39]	9	5	—	25	33	—	17	8.3	8.3	—	—
8.	Tetanus	*Clostridia tetani*.	[40]	1	0.6	—	100	—	—	—	—	—	—	—
9.	TB	*Mycobacterium tuberculosis*.	[41]	27	16	12	32	44	4	4	—	4	—	—
10.	Typhoid	*Salmonella typhi*.	[42]	10	5.8	—	40	10	—	20	10	10	10	—

Abbreviations: Bu, bulb; L, leaf; La, latex; No, number; R, root; Rh, rhizome; Sd, seed; St, stem; Wp, whole part.

**Table 4:** Medicinal plant species, family, local name, parts used, disease treated, mode of preparation, route of administration, application, and citation.

Medicinal plant species list	Family	Local name	Parts used	Disease treated	Mode of preparation	Citation
1. *Acacia abyssinica* Hochst. ex Benth.	Fabaceae	Laaftoo (Or)	L	TB	Warming the fresh leaf and rubbing the swelling dermally.	[43]
		Girar (Am)	Sd	Syphilis	Mixing the seed powder with water and drunk orally.	[44]
2. *Acacia etbaica* Schweinf.	Fabaceae	Girar (Am)	R	Diarrhea	Mixing the root powder with water, drink in a cup orally.	[45]
			NM	Gonorrhea	NM.	[46]
		Doddotii (Or)	L	Gonorrhea	The fresh leaves of the *Vachellia etbaica* is chewed and crushed, boiled, and drunk.	[47]
3. *Acanthospermum hispidum* DC.	Asteraceae	Qummuxxo (Or)	L	Tetanus	Pounding the leaf and putting the powder on the affected part dermally.	[48]
4. *Achyranthes aspera* L.	Amaranthaceae	Darguu (Or)	R	Chancroid	Pound the root, boil it with water and drink orally.	[48]
		Begegechoo (Koorete)	St, F	Gonorrhea	Combine the green fruit and stem with butter and drink for 7 days.	[49]
		Telenji (Am)	R	Diarrhea	Powder of root mixed with water is given orally.	[58]
5. *Agarista salicifolia* (Lam.) G.Don	Ericaceae	Sotra (Or)	Ba	Bone TB	Fresh bark is crushed and extract is applied dermally.	[50]
6. *Ajuga integrifolia* Buch.‐Ham. ex D.Don	Lamiaceae	Akembiye (Am)	Wp	Diarrhea	Adding the whole part to hot water, filter and drink with tea glass.	[48]
		—	Wp	Pneumonia	Crushing the whole parts and filtering the liquid and drink.	[51]
7. *Acokanthera schimperi* (A. DC.) Schweinf.	Apocynaceae	Qaraaruu (Or)	L	Leprosy	Crushing the leaf and adding dermally.	[52]
8. *Allium sativum* L.	Amaryllidaceae	Nech shinkurt (Am)	Bu	Typhoid	Dried or fresh bulb with *Allium cepa* and *Nigella sativa* is soaked in water, filtered and given orally.	[53]
		Nech shinkurt (Am)	Sd	Diarrhea	The seed is chewed and swallowed orally.	[44]
9. *Aloe macrocarpa* Tod.	Asphodelaceae	Argiisa (Or)	L	Leprosy	Fresh leaves are pounded and mixed with butter and painting on the skin dermally.	[43]
10. *Amaranthus caudatus* L.	Amaranthaceae	Bahr tef (Am)	L	Diarrhea	Squeezing the leaves and make a juice, drink the juice orally.	[54]
		Iyyaasuu (Or)	L	Diarrhea	Pounding the dry leaf and boiling and drink orally.	[55]
11. *Anogeissus leiocarpa* (DC.) Guill. and Perr.	Combretaceae	Hanse (Tig)	Ba, L	Diarrhea	NM.	[56]
		Kekera (Am)	Sb	Diarrhea	Drinking the stem bark decoction.	[57]
12. *Argemone mexicana* L.	Papaveraceae	Kossalae (Gedio)	L	Diarrhea	Powdered fresh leaf mixed with residue of local “tella (in Amharic)” or “areqie” (local liquer, in Amharic) is given orally.	[58]
13. *Aristolochia involuta* X.X.Zhu, Z.X.Ma and J.S.Ma	Aristolochiaceae	Abujenajil (Be)	Sd	Diarrhea	Pounding the seed and mixing with water and drink.	[59]
14. *Artemisia absinthium* L.	Asteraceae	Arity (Am)	Wp	Syphilis	Powdered, mixed in water and drunk orally.	[60]
		Arity (Am)		Diarrhea	Boil leaves with water and drink half cup of coffee of the hot decoction every day.	[54]
15. *Artemisia abyssinica* Sch.Bip. ex Oliv. and Hiern	Asteraceae	Chikugn (Am)	R, L	Diarrhea	Root/leaf crushed and mixed with water then drink.	[61]
16. *Artemisia afra* Jacq. ex Willd.	Asteraceae	Chiyanchiy (Gur)	St, L	Gonorrhea and syphilis	Pounding the stem and leaf of *Alistema afra*, mixing with little water and drink every morning for 3 days.	[62]
17. *Asparagus africanus* Lam.	Asparagaceae	Saritii (Or)	R	Chancroid	Grinding the root, boiling it with water and drinking it orally.	[48]
18. *Arundinaria alpina* K. Schum	Poaceae	Leman (Or)	L	Diarrhea	Leaves are pounded and squeezed and mixed with water and drink.	[50]
19. *Balanites aegyptiaca* (L.) Delile	Balanitaceae	Mekie (Tig)	F	TB	NM.	[56]
		Uddayto (Af)	R	Diarrhea	Crushing and mixing with water and shaking then drink.	[63]
		Alayto (Af)	L, Ba	Dysentery	Taken orally (MPNM).	[64]
20. *Barleria eranthemoides* R.Br. ex C.B.Clarke	Acanthaceae	Yeset Af (Am)	R	Diarrhea	NM.	[65]
21. *Bersama abyssinica* Fresen.	Francoaceae	Koraqa (Or)	L	Diarrhea	Crush or pound, mix it with water, and drink orally.	[50]
		Azamir (Am)	L, St	Diarrhea	NM.	[66]
22. *Bidens pilosa* L.	Asteraceae	Chigogot (Am)	R	Diarrhea	The root is pounded and mixed with water, then drunk orally.	[44]
23. *Boscia coriacea* Graells	Capparaceae	Homura/Aytneba (Af)	L	Leprosy	Applied dermally.	[64]
24. *Boswellia papyrifera* (Del.) Hochst.	Burseraceae	Meker (Tig)	Ba	Diarrhea	NM.	[56]
25. *Brucea antidysenterica* J.F.Mill.	Simaroubaceae	Abalo (Am)	R, L	Bloody diarrhea	Pound the dry root and dissolve in water, or the leaf is squeezed and drink 1/2 of coffee cup of the juice.	[43]
			R	Diarrhea	Root powdered and mixed in water and drunk.	[47]
			Ba	Diarrhea	The bark powder will be mixed with milk and then drunk orally.	[44]
26. *Buddleja polystachya* Fresen.	Scrophulariaceae	Adaaddo (Or)	Sd	Diarrhea	The dried seeds are pounded, mixed with honey and eaten orally.	[43]
27. *Cadaba glandulosa* Forssk.	Capparaceae	Udodoita (Af)	L	Diarrhea	Taken orally.	[67]
28. *Calpurnia aurea* (Aiton) Benth.	Fabaceae	Digta (Am)	S	Diarrhea	Crush the seed with milk and then drink.	[68]
				TB	NM.	[56]
			L, Sd	Diarrhea	Taken orally.	[69]
			Sd, L	Diarrhea	Chewing and swallowing the seed. Leaf is crushed and the liquid is drunk.	[70]
			L	Typhoid	NM.	[69]
			L	Diarrhea	Fresh leaf soaked in water and drink orally.	[53]
			F	Diarrhea	Crush, pound, and drunk orally.	[60]
			R	Gonorrhea	Fresh root is crushed, mixed with water, and one cup is taken for 3 days orally.	[71]
			L	Diarrhea	Fresh leaf is crushed, soaked in water for 2–3 h, decanted, and one glass is administrated orally.	[71]
			L, R	Diarrhea	Crush the leaf and root then add in boiled water, and drink the mixed liquid at the morning before food orally.	[44]
29. *Capsicum annuum* L.	Solanaceae	Qaria (Am)	F	Dysentery	Its fruits with *A. sativum*, *Zingiber officinale*, and *Nigella sativum* are immersed in water for 2–3 days and drink continuously.	[72]
30. *Carica papaya* L.	Caricaceae	Papaya (Am)	L	Pneumonia	Pounding the leaf and drinking it or boiling the leaf and fumigate the steam, oral and nasal.	[59]
		—	R	Diarrhea	By grinding the roots and mixed with water and drink it orally.	[59]
31. *Carissa spinarum* L.	Apocynaceae	Agam (Am)	L	Diarrhea	Pounding the leaf and mixing with *coffee* and drink.	[73]
		—	R	Gonorrhea	Crushing the root and mixing it with water, then, drink one cup for 5 days.	[43]
		—	L	Diarrhea	Crush the leaf and smash, then, drink one cup for 3–5 days.	[43]
		—	Sb, R	Gonorrhea	Dried stem bark powder and fresh root decoction are mixed with honey and drink.	[44]
		—	R	Diarrhea	Boiling the root and drink.	[74]
		—	R	Diarrhea	Dry root is pounded and powdered; salt is added; it is made as a solution, and drink orally.	[49]
		—	L	Diarrhea	The powdered leaf is blended with coffee and subsequently boiled, after which one cup is consumed until recovery.	[75]
32. *Catha edulis* (Vahl) Forssk. ex Endl.	Celastraceae	Chat (Am)	L	Diarrhea	Chewing the leaf mixed with honey coffee or sugar 50 g for 3 days.	[43]
33. *Cissampelos pareira* L.	Menispermaceae	Abujelajil (Be)	R	Diarrhea	Grind and drink with water orally.	[41]
34. *Clerodendrum myricoides* (Hochst.) R.Br. ex Vatke	Lamiaceae	Tiiro (So)	L	Diarrhea	Crush and mix with water, drink orally.	[76]
35. *Cissus quadrangularis* L.	Vitaceae		St	Leprosy	Applied dermally and orally (MPNM).	[52]
36. *Citrus* × *aurantiifolia* (Christm.) Swingle	Rutaceae	Lomi (Am)	F	Diarrhea	Adding sugar and salt.	[68]
			F	Gonorrhea	Crushing and oral.	[77]
37. *Clutia abyssinica* Jaub. and Spach	Peraceae	Fiyele‐fej (Am)	St	Dysentery	Crush fresh stem making it powder and then drink orally.	[78]
38. *Coccinia abyssinica* (Lam.) Cogn.	Cucurbitaceae	Ancootee (Or)	R	TB	NM.	[79]
			R	TB	Cooking the root with the leaves of *Croton macrostachyus* and eaten with “injera” (thin bread) for 4 days.	[80]
39. *Coffea arabica* L.	Rubiaceae	Buna (Am)	L	Diarrhea	The roasted and grind powder mixed with honey is eaten.	[81]
		—	Sd	Diarrhea	The powder is mixed with honey and eaten.	[61]
		—	Sd	Diarrhea	The seed of *C. arabica* is roasted, crushed, powdered, boiled; and filtrate one cup of tea, mixed with few drop of oil then drink.	[43]
		—	Sd	Diarrhea	Dried, grind, and mixed with water, taken orally.	[63]
		—	Sd	Diarrhea	Dry seed is roasted, powdered, mixed with honey, and one or two spoons is taken in the morning time for 3 days.	[49]
		—	Sd	Diarrhea	Roast the seed, pound and mix with honey then swallow it	[82]
		—	Sd	Diarrhea	Roast the dried coffee bean and powder, then give it to the patient by mixing with honey.	[80]
40. *Colocasia esculenta* (L.) Schott.	Araceae	Godarre (Or)	Rh	Diarrhea	Crush/pound dry/fresh concocted with *Z*. *officinale* rhizome is taken with coffee as drink.	[45]
41. *Commicarpus helenae* (Schult.) Meikle	Nyctaginaceae	—	WP	Typhoid	Applied orally and nasally (MPNM).	[52]
42. *Conyza bonariensis* (L.) Cronquist	Asteraceae	Qilqilia (Am)	L	Diarrhea	Applied orally.	[77]
43. *Cordia africana* Lam.	Boraginaceae	Wanza (Am)	L	Diarrhea	Crush leaves with cold water and drink the fluid.	[68]
		—	R, Rb	Diarrhea	Applied orally.	[69]
		Wodeessa (Or)	F	Diarrhea	Eating 5–7 fruits daily in the morning.	[50]
		—	St	Gonorrhea	Pounding fresh bud and mix with water and butter, filter, and drink the liquid orally.	[55]
		—	Ba	Diarrhea	Crushing bark, mix with water and drink at morning.	[44]
44. *Croton macrostachyus* Hochst. ex Delile	Euphorbiaceae	Bisana (Am)	L	Gonorrhea	Pound the leaves and mix with water and butter and filter and drink orally.	[75]
		Bakkanniisa (Or)	L	Gonorrhea	Cook in fresh leaf tips and drink two spoon of liquid for 5 consecutive days orally.	[43]
		—	L	Gonorrhea	Applied orally (MPNM).	[69]
		—	L	Diarrhea	Powder shade‐dried leaves, mix with hot water, and the mixture taken orally.	[49]
		—	R	Gonorrhea	Chewing and swallowing the root orally.	[62]
		—	F, L	TB	Pounding the leaf, form liquid, chewing, boiling and drink oral.	[83]
		—	L	Diarrhea	Leaf powder mixed with water is taken orally.	[84]
45. *Cuminum cyminum* L.	Apiaceae	Kemmuuna (Or)	L	Chancroid	Pounding the leaf with *Tragia cinerea* leaf, mix with water, and drink it orally.	[48]
46. *Cucumis dipsaceus* Ehrenb. ex Spach	Cucurbitaceae	Hafaflo (Tig)	R	TB	NM.	[56]
47. *Cucumis ficifolius* A.Rich	Cucurbitaceae	Yemidirembuay (Am)	R	Diarrhea	The root is crushed and mixed with water before being allowed to drink.	[45]
		—	R	Diarrhea	Squeezing. The root dug out with horn handled knife and squeezed. One spoon of the filtrate can be given for children.	[85]
		—	F	TB (lung)	Mixture of dried fruit, *Gnidia glauca* root and *A. sativum* bulb soaked with local beer is given orally.	[53]
		—	R	Diarrhea	The root is dried and pounded then mixed with tella (ale) and drink.	[44]
48. *Cucumis pustulatus* Naudin ex Hook.f.	Cucurbitaceae	Haadhatu (Or)	R	TB	Chewing the root or crushing the root, making solution and drinking one coffee cup daily until cured.	[81]
49. *Cucurbita pepo* L.	Cucurbitaceae	Buqqee (Or)	Sd	Gonorrhea	Pounded, mixing with water and filter one tea cup a day for a week.	[46]
50. *Cynoglossum coeruleum* Hochst. ex A. DC.	Boraginaceae	Fkrutena (Am)	R, Sd	Syphilis	Pound the seed and root and mix with vaseline and then cream on the affected part.	[82]
51. *Cynoglossum amplifolium* Hochst. ex A.DC.	Boraginaceae	—	L, R	Diarrhea	Take orally (MPNM).	[86]
52. *Cyphostemma adenocaule* (Steud. ex A.Rich.) Desc. ex Wild and R.B.Drumm	Vitaceae	Aserkush Tebetebkush (Am)	L	Syphilis	NM.	[65]
53. *Dodonaea angustifolia* L.f.	Sapindaceae	Kitkita (Am)	L	Dysentery	Soak the leaf in water then filter. The filtrate is given orally with sugar.	[45]
			L	Dysentery	Fresh leaf soaked with water and sugar is given orally.	[53]
			L	Diarrhea	Fresh leaf soaked in water for some hours is given for drinking.	[55]
		Ittancha (Sd)	L	Diarrhea	Boiling, grinding, squeezing.	[51]
54. *Dorstenia barnimiana* Schweinf.	Moraceae	Work bemeda (Am)	R	Leprosy	Prepare a local liquer (Areke in Amharic), from the mix of crushed root, seeds of *Lepidium sativum* L. and *sorghum* flour, drink it until cured.	[54]
			R	Leprosy	Taken orally (MPNM).	[69]
			R	Syphilis	Root powder is taken with honey orally in the morning.	[84]
			R	Diarrhea	Pounding the root, mix with honey and ferment for 7 days, drink orally in the morning until cured.	[84]
55. *Ehretia cymosa* Willd. ex Roem. and Schult.	Boraginaceae	Uraaga (Or)	L	Gonorrhea	Crushing its leaves with the leaves of *Fagaropsis angolensis*, *Acmella caulirhiza,* and internal part of stem bark of *C. macrostachyus*, making solution and drink one water glass at once.	[81]
56. *Ekebergia capensis* Sparrm.	Meliaceae	Lol (Am)	Ba	Syphilis	Soaking the fresh bark with root of *C. ficifolius* for 3 days and drink the infusion for a week with a cup of coffee.	[74]
57. *Embelia schimperi* Vatke.	Primulaceae	Sharrengo (Ama)	R	Leprosy	Crushing fresh root with water, drink for several days.	[58]
58. *Ensete ventricosum* (Welw.) Cheesman	Musaceae	Koba (Am)	St, R	Diarrhea	Specific varieties eaten.	[75]
		Esset (Gur)	R	Gonorrhea, syphilis	The of root of *E. ventricosum* is cooked and eat with yoghurt.	[62]
59. *Eucalyptus globulus* Labill.	Myrtaceae	Bahr zaf (Or)	L	Typhoid	Fresh leaves concocted, crushed, mixed with water and filtered orally.	[78]
60. *Euclea racemosa* subsp. *Schimperi* (A. DC.) F. White	Ebenaceae	Me′essaa (Or)	R	Chancroid	Boiling the root of *E. racemosa* with water and drink it orally.	[48]
61. *Euphorbia abyssinica* J.F.Gmel.	Euphorbiaceae	Qulkual (Am)	La	Gonorrhea	Mixing the latex with powder of *Eragrostis tef* and drink it.	[87]
62. *Euphorbia ampliphylla* Pax	Euphorbiaceae	Hadaama (Or)	La	Gonorrhea	Taking some amount of the latex, cooking it with Qocho (*E. ventricosum*) bread and eating it once for all.	[81]
63. *Euphorbia cryptospinosa* P.R.O.Bally	Euphorbiaceae	Aananno (Or)	R	TB	Crush the root with the root of *Solanum incanum* and *Osyris quadripartita*, making solution and adding hone, drink orally.	[81]
64. *Euphorbia depauperata* Hochst. ex A.Rich.	Euphorbiaceae	Gurii (Or)	R	Gonorrhea	Crush fresh root, decocting and drink orally.	[78]
65. *Euphorbia dumalis* S.Carter	Euphorbiaceae	Gurii (Or)	Sb	Gonorrhea	Pound and mix with water and honey and drink orally.	[78]
66. *Euphorbia lathyris* L.	Euphorbiaceae	Ambuluk (Or)	Sd	Gonorrhea	Pounding or crushing, mixing water, drink orally.	[78]
67. *Euphorbia schimperiana* Scheele	Euphorbiaceae	Guurii (Or)	St	Gonorrhea	Pounding or crushing, decocting, applying dermally.	[50]
68. *Euphorbia tirucalli* L.	Euphorbiaceae	Qinchib (Am)		TB	Dry form of the plant.	[73]
69. *Ficus sur* Forssk.	Moraceae	Shola (Am)	La	Dysentery	About one spoon of sap is taken orally.	[72]
70. *Ficus sycomorus* L.	Moraceae	Qiltu (Or)		Diarrhea	The crushed and squeezed fresh bark of the plant is taken orally.	[50]
71. *Ficus thonningii* Blume.	Moraceae	Chibha (Am)	R	Diarrhea	Chewing root.	[84]
72. *Foeniculum vulgare* Mill.	Apiaceae	Ensilal (Am)	L	Gonorrhea	Fresh or dried, concocted, crushed, and taken orally.	[78]
		NM	L	Gonorrhea	Boiling the leaves and drink when there is such problem.	[62]
73. *Gardenia ternifolia* Schumach. and Thonn.	Rubiaceae	Gambeela (Or)	Ba	Gonorrhea	Crushing with garlic, mix with water and one tea cup of filerate is taken three times/day for a week.	[46]
74. *Gladiolus dalenii* Van Geel	Iridaceae	Kelede (Or)	R	Gonorrhea	Fresh or dried, crushed, mixed with water and drink orally.	[78]
75. *Gnidia involucrata* Steud. ex A.Rich.	Thymelaeaceae	Bortoo (Or)	R	Gonorrhea	Crush the root, drink one water glass at once.	[81]
76. *Gomphocarpus purpurascens* A. Gray	Asclepiadaceae	Ari‐Yuyo (Or)	R	Pneumonia	Fumigate the root and/or boiling and drink it, orally or nasally.	[48]
77. *Grewia erythraea* Schweinf	Malvaceae	Hidayto (Af)	L	Typhoid	Oral (MPNM).	[64]
		Hidayto (Af)	R	Leprosy	Applied dermally (MPNM).	[64]
78. *Grewia villosa* Willd.	Malvaceae	Bururi (Koorete)	L	Diarrhea	Juice of leaves is taken orally.	[49]
		Bururi (Koorete)	L	Gonorrhea	Dried and powdered leaves are mixed with melted butter and drunk.	[49]
79. *Hagenia abyssinica* (Bruce) J.F.Gmel.	Rosaceae	Koso (Am)	Sd	Diarrhea	Powdered seed mixed with a cup of coffee and drink.	[88]
80. *Heliotropium cinerascens* Aitch.	Boraginaceae	NM	L	Leprosy	Apply topically/dermally (MPNM).	[64]
81. *Hordeum vulgare* L.	Poaceae	Gebis (Am)	Sd	Diarrhea	Seeds are immersed in water and allowed to germinate before being dried, roasted, and pulverized. The powder is then heated in water and drunk until the pain cures.	[45]
82. *Impatiens ethiopica* Grey‐Wilson	Balsaminaceae	Ensosla (Am)	R	Gonorrhea	Fresh root crushed and applied dermally.	[78]
83. *Impatiens tinctoria* A. Rich.	Balsaminaceae	Angabisha (Or)	R	Gonorrhea	Crush fresh root, or pound the dry root, mix with water and the drink.	[70]
84. *Indigofera oblongifolia* Forssk.	Fabaceae	NM	L, R	Dysentery	Taken orally (MPNM)	[64]
85. *Indigofera spicata* Forssk.	Fabaceae	NM	R	Diarrhea	Taken orally (MPNM).	[86]
86. *Jasminum abyssinicum* Hochst. ex DC.	Oleaceae	Tenbelel (Am)	NM	Diarrhea	NM.	[89]
87. *Jatropha glauca* Vahl	Euphorbiaceae	Qablis (Af)	R	TB	The infusion of fresh root is administered nasally and orally.	[90]
88. *Justicia schimperiana* (Hochst. ex Nees) T. Anderson	Acanthaceae	Dhummugaa/Smiza (Or)	R	Gonorrhea	Root is crushed, boiled, add cow milk and drunk orally.	[43]
		Sensel, Simiza (Am)	L	Typhoid	NM.	[65]
89. *Juniperus procera* Hochst. ex Endl.	Cupressaceae	Honicho (Sd)	L	Diarrhea	Pounding the leaf.	[51]
90. *Kalanchoe petitiana* A. Rich.	Crassulaceae	Hancuule (Or)	L, St	Pneumonia	The stem and leaf are heated on fire, apply on the place dermally.	[70]
		Bosoqee (Or)	L	Gonorrhea	Fresh leaf juice is applied on the wound.	[55]
91. *Kanahia laniflora* (Forssk.) R.Br.	Apocynaceae	Leehamohcaxa (Af)	L	TB	The infusion of fresh leaves is administered nasally, and a small amount is drunk.	[90]
92. *Kniphofia isoetifolia* Hochst.	Asphodelaceae	Shinshile (Or)	R	Gonorrhea	Fresh or dried concocted, crushed, oral powdered, mixed with coffee and sugar.	[78]
93. *Lagenaria siceraria* (Molina) Standl.	Cucurbitaceae		F	Gonorrhea	Ripe fruits including seeds are immersed in water for overnight; the water is taken orally in the morning before breakfast.	[58]
94. *Lantana camara* L.	Verbenaceae	Yewef kollo (Am)	St	Diarrhea	Dry stem powder mixed with water is taken orally.	[58]
95. *Lepidium sativum* L.	Brassicaceae	Feto (Am)	Sd	Diarrhea	Grind seeds into paste‐like food and then eaten or mixed with butter and water and drunk.	[85]
			Sd	Dysentery	The seed is pounded, mixed with yoghurt; shaken well and drunk.	[58]
			Sd	Diarrhea	Dry seeds are pounded, powdered, mixed with water, and the solution has to be taken orally.	[71]
96. *Leonotis ocymifolia* (Burm F.) A. Iwarsson.	Lamiaceae	Feres zeng (Am)	F, L	Diarrhea	Powder of dried fruit and leaf is mixed with honey and then given orally.	[85]
			L	Diarrhea	Dried leaf and fruit powder mixed with honey is given orally.	[86]
			L, F	Diarrhea	Mixing the dried leaf and fruit powder with honey, drink orally.	[55]
97. *Leucas deflexa* Hook.f.	Lamiaceae		L	Diarrhea	Oral (MPNM).	[86]
98. *Linum usitatissimum* L.	Linaceae	Telba (Am)	Sd	Diarrhea	The powder will be boiled, then drink like soup, oral.	[61]
99. *Lycopersicon esculentum* (L.) Mill	Solanaceae	Timatim (Am)	L	Gonorrhea	Fresh or dried concocted, crushed, oral decocted.	[78]
100. *Malva parviflora* Huds.	Malvaceae	Siito (Or)	L	TB	The leaf is crushed; powder is mixed with water and drunk.	[70]
101. *Malva verticillata* L.	Malvaceae	Yewusha nacha (Am)	R	Dysentery	Dried root with *C. aurea* root soaked with water is given orally.	[53]
102. *Maesa lanceolata* G.Don	Myrsinaceae	Kilaba (Am)	L	Leprosy	Dried or fresh leaf with *Clematis simensis* leaf powder mixed with butter is applied topically.	[53]
		—	L, St	Diarrhea	NM.	[66]
		—	R	Diarrhea	Dried root together with *C. aurea* root soaked in water for hours is given orally.	[55]
103. *Mangifera indica* L.	Anacardiaceae	Mango (Am)	Ba	Diarrhea	Fresh bark is crushed and mixed with water, drink.	[85]
104. *Melia azedarach* L.	Meliaceae	Nim (Am)	L	Diarrhea	Fresh.	[52]
105. *Myrtus communis* L.	Myrtaceae	Adasii (Or)	L	Pneumonia	Boiling the leaf and fumigating the steam, nasally.	[48]
106. *Nicotiana tabacum* L.	Solanaceae	Tombowae (Koorete)	L	Diarrhea	Crushed, decocted, and concocted fresh leaves are taken, and powdered roots are mixed with water or milk and drunk.	[49]
		—	L	Gonorrhea	Mixing the leaf with hot water and drink.	[49]
107. *Nigella sativa* L.	Ranunculaceae	Tikur Azmud (Am)	Sd	Pneumonia	One spoon of *N*. *sativa* (cumin) oil was taken morning and evening.	[70]
108. *Ocimum americanum* L.	Lamiaceae	Ziqaqibie (Am)	L	Dysentery	Leaf is pounded, mixed with water, and drunk.	[72]
109. *Ocimum gratissimum* L.	Lamiaceae	Damakase (Am)	L	Diarrhea	Pound the leaves and extract the juice and drink.	[75]
			L	Diarrhea	Fresh leaf is boiled with tea and one cup of tea is drunk, oral.	[71]
110. *Olinia rochetiana* A. Juss.	Penaeaceae	Gunaa (Or)	L	Bone TB	Fresh leaf is crushed and macerated with water and drink.	[50]
			L	Diarrhea	Fresh leaf is crushed and macerated with water.	[50]
111. *Osyris quadripartita* Salzm. ex Decne.	Santalaceae	Waatoo (Or)	R	TB	Pounding, making solution and drink one water glass daily for a month.	[81]
112. *Otostegia integrifolia* Benth	Lamiaceae	Tenjut (Am)	L, R	Pneumonia	Grind the dried leaves, then blend with *Ximenia americana* seed oil, and gently massage onto the hair for 3 consecutive days.	[83]
113. *Pavonia patens* (Andrews) Chiov.	Malvaceae	Hiccini (Or)	L	Diarrhea	Pounding the leaf with *Calpurnia auria* leaf, mix with water and drink it orally.	[48]
114. *Pentas lanceolata* (Forssk.) Deflers	Rubiaceae	Cunfaa (Or)	L	Gonorrhea	Pounding the leaves with the leaves of *F. angolensis*, *A. caulirhiza,* and internal stem bark of *C. macrostachyus*, making solution, adding honey and drink one water glass at once.	[81]
115. *Phyllanthus pseudoniruri* Müll. Arg.	Euphorbiaceae		R	Bloody diarrhea	The root is crushed and mixed with coffee and drunk.	[75]
116. *Phytolacca dodecandra* L′ Her.	Phytolaccaceae	Andoodee (Or)	R	Gonorrhea	Pounding the root and mix with water and drink for 2 days.	[47]
117. *Piliostigma thonningii* (Schumach.) Milne‐Redh	Fabaceae	Mekel (Be)	R	Bloody diarrhea	Grind, dispersed in water, and drunk.	[59]
118. *Plectranthus lactiflorus* (Vatke) Agnew	Lamiaceae	Dibrk (Am)	L, R	Diarrhea	Roots and leaves are crushed; mixed with water and the filtrate is drunk.	[82]
119. *Plumbago zeylanica* L.	Plumbaginaceae	Amira (Am)	L	Bone TB	Taken orally (MPNM).	[69]
120. *Podocarpus falcatus (*Thunb.) Endl.	Podocarpaceae	Birbirsa (Or)	L	Diarrhea	Chop the leaves, mix with water and drink one coffee cup 2 times a day for 3 days.	[81]
121. *Polygala sphenoptera* Fresen.	Polygalaceae	Harmel (Or)	R	Chancroid	Boil the root with water and drink orally.	[48]
122. *Protea gaguedi* J.F.Gmel	Proteaceae	Daansee (Or)	L	Diarrhea	Chopping the leaf with the leaf of *F. angolensis* and fruits of *S. incanum* mixing with water drink orally.	[81]
123. *Prunus africana* (Hook.f.) Kalkman	Rosaceae	Oomii (Or)	Ba	Pneumonia	Crushed and boiled and then filtered one glass/day for a week.	[46]
		Sukkee (Or)	L	Diarrhea	Pound the leaves with the leaves of *Clematis hirsuta*, *C. aurea*, *Ehretia obtusifolia*, *C. macrostachyus,* and *Teclea simplicifolia*, mix with water, and drink one glass of water.	[81]
124. *Pseudocedrela kotschyi* (Schweinf.) Harms	Meliaceae	Aduruba (Be)	Ba	Diarrhea	Eaten as it is.	[59]
125. *Psidium guajava* L	Myrtaceae	Zeytun (Am)	F	Dysentery	Amoebic dysentery can be treated using the fruit.	[45]
		Zeyituna (Or)	R	Dysentery	Grind the roots *of P. guajava* and *Impatiens rothii,* mix with water, and drink it orally.	[54]
126. *Pterolobium stellatum* (Forssk) Brenan	Fabaceae	Qudu (Be)	R	Diarrhea	The root (after removing the cover) is boiled together with *Acacia* sp. in water and drunk.	[59]
127. *Rhamnus prinoides* L′Her.	Rhamnaceae	Gesho (Am)	L	Dysentery	Fresh leaves are squashed and mixed with water, and about one cup is drunk.	[72]
128. *Rhus vulgaris* Meikle	Anacardiaceae	Kammo (Am)	F	TB (Lung)	Dried fruit powder is given orally before food.	[53]
129. *Ricinus communis* L.	Euphorbiaeae	Qobbo (Or)	NM	TB	Fresh part (MPNM).	[52]
		Qobbo (Or)	NM	TB	Fresh, topical (MPNM).	[52]
130. *Rubia cordifolia* L.	Rubiaceae	Anqis (Or)	R	Diarrhea	Fresh or dried powdered, decocted and oral.	[78]
131. *Rumex abyssinicus* Jacq.	Polygonaceae	Meqmeqo (Am)	R	TB	NM.	[56]
		Dhangagoo (Or)	R	Gonorrhea	Pounding the roots, boiling, adding butter, and drinking one water glass daily until cured.	[81]
		—		Gonorrhea	Crushed, pounded, and boiled, drunk orally.	[50]
		—		Diarrhea	Crushed or pounded, boiled with water, and drink orally.	[50]
132. *Rumex nepalensis* Spreng.	Polygonaceae	Shaabee (Or)	L	Diarrhea	Fresh leaf crushed, mixed with water, and drunk orally.	[50]
		—	R	Diarrhea	The crushed root will be mixed with water, then drunk the filtrated juice part orally.	[44]
133. *Ruta chalepensis* L.	Rutaceae	Tenadam (Am)	Sd	Diarrhea	The pounded seed will be mixed with coffee then drunk orally.	[61]
		—	L	Diarrhea	Fresh leaf together with salt (concoction) is chewed orally.	[71]
		—	Sd	Diarrhea	Seed is pounded and mixed with yogurt and drunk orally.	[44]
134. *Rytigynia neglecta* (Hiern) Robyns	Rubiaceae	Gaaloo (Or)	R	Pneumonia	Fresh root is crushed and boiled in water, oral.	[50]
135. *Salvadora persica* L.	Salvadoraceae	Qadayto (Af)	R	TB	The infusion of the root, the root of *Tamarix aphylla* and the leaves of *Aloe* sp. is administered orally.	[86]
136. *Salvia nilotica* Juss. ex Jacq.	Lamiaceae	Shokoksa (O)	F	Diarrhea	The fruit juice is taken orally.	[49]
137. *Schinus molle* L.	Anacardiaceae	Kundo berbere (Am)	L	TB	Crushed seeds are mixed with honey and then eaten.	[54]
138. *Senna didymobotrya* (Fresen.) H.S.Irwin and Barneby	Fabaceae	Yeferenjdgta (Am)	Sd	Diarrhea	The seed is crushed and roasted, then drunk with coffee.	[45]
		Sanamiki (Or)	L	Gonorrhea	Pound the leaf, mix with water, boil, and drink.	[70]
139. *Senna occidentalis* (L.) Link	Fabaceae	Assenmeka (Ama)	R	Gonorrhea	Fresh root powder with honey is taken as a drink for before sexual intercouse.	[58]
140. *Salvia schimperi* Benth	Lamiaceae	Senemekii (Or)	L	Diarrhea	Pound, mix with water and drink one glass/day for a week.	[46]
141. *Senra incana* Cav.	Malvaceae	—	L	Diarrhea	Pound the leaf, mix with water, and drink it in a cup of water after shaking.	[85]
142. *Sida schimperiana* Hochst. ex A. Rich.	Malvaceae	Chifrig (Am)	NM	TB	Fresh part.	[73]
			L	Dysentery	The dried leaf is grind and mixed in a cup of water. Drink two spoon solutions after shaking.	[72]
143. *Solanum incanum* L.	Solanaceae	Embuay (Am)	R	Gonorrhea	Dried root powder mixed with water and sugar is drunk.	[49]
144. *Solanum somalense* Franch.	Solanaceae	Yeshehochu Kitel/Shejerete Jin (Am)	L	Diarrhea	NM.	[65]
		NM	St	Typhoid	Topical, nasal (MPNM).	[64]
145. *Sorghum bicolor* (L.) Moench	Poaceae	Mashilla (Am)	Sd	Diarrhea	Dried seed is powdered and a thin pancake—like fermented bread is prepared and is eaten.	[75]
146. *Stephania abyssinica* (Quart.‐Dill. and A.Rich.) Walp.	Menispermaceae	Kalala (Or)	R	Gonorrhea	Fresh root crushed, decocted and drunk orally.	[78]
147. *Stereospermum kunthianum* Cham.	Bignoniaceae	Estegne eyo (Be)	R	Diarrhea	Grind, dispersed in water and drunk.	[59]
		Zana (Am)	Sb	Diarrhea	Oral (MPNM).	[69]
148. *Syzygium guineense* (Willd.) DC.	Myrtaceae	Dokima (Am)	Sb/Rb	Diarrhea	Mix the powder with honey/water, and then drink orally.	[57]
		—	L	Leprosy	Dried leaf powder mixed with honey, applied for 3 days.	[87]
		—	St	Gonorrhea	Juice from stem is taken for sexual diseases.	[49]
		—	L	Diarrhea	Fresh leaf is chewed and ingested, orally.	[50]
149. *Syzygium aromaticum* (L.) Merr. and L.M.Perry	Myrtaceae	Qernfud (Am)	F	Diarrhea	Consume the fruit with a cup of coffee.	[83]
150. *Tagetes minuta* L.	Asteraceae	Hada Gola (Or)	L	Diarrhea	Fresh or dried concocted, crushed, decocted and drink.	[78]
151. *Tamarindus indica* L.	Fabaceae	Mala (Be)	F	Diarrhea	Chopped, dispersed in water, and the suspension is drunk.	[59]
		Hura (Af)	Ba	Typhoid	Oral (MPNM).	[64]
152. *Tamarix aphylla* (L.) H.Karst	Tamaricaceae	Saaganto (Af)	R	TB	The infusion of the root, the root of *T. aphylla* and the leaves of *Aloe* sp. is administered orally.	[86]
153. *Tapinanthus globifer* (A.Rich.) Tiegh	Loranthaceae	Hafa‐teketsila (Am)	Wp	Gland TB	Dermal (MPNM).	[69]
154. *Tephrosia elata* Defers.	Fabaceae	Yeait‐hareg (Am)	R	Pneumonia	Pound the dried root, then put the powder on fire, then sniffled the fumigate.	[83]
155. *Terminalia brownii* Fresen.	Combretaceae	Weyba (Am)	Ba, L	Diarrhea	NM.	[56]
156. *Thymus schimperi* Ronniger	Lamiaceae	Tosign (Am)	L, R	Gonorrhea	Dried leaves and roots are pounded and drunk with tea, oral.	[44]
157. *Trigonella foenum-graecum* L.	Fabaceae	Dinbilal (Am)	Sd	Typhoid, pneumonia	The seed is crushed, powdered, mixed with lemon liquid and water; drink the juice; the powder is mixed with cheese for colds.	[70]
158. *Urtica simensis* Hochst.ex A.Rich.	Urticaceae	Sama (Am)	R	Gonorrhea	Infusion of root is prepared and the wash genital organ with it once daily.	[74]
			R, L	Gonorrhea	The root and leaves of this plant crushed and mixed with powder of wheat or barley then cooked and eat continuously for 7 days.	[62]
			R, L	Gonorrhea	Powder, mix in water and drunk the filtrate orally.	[60]
		Dobbii (Or)	R, L	Gonorrhea	Pounding, topical (MPNM).	[52]
159. *Verbascum sinaiticum* Benth.	Scrophulariaceae	Yeahya joro (Am)	L	Diarrhea	NM.	[66]
			R	Diarrhea	Juice of root is taken orally.	[84]
160. *Verbena officinalis* L.	Verbenaceae	Atuch (Am)	R, St	Diarrhea	Pound the leaf, stem, and root and mix with water then drunk.	[82]
			R, L	Diarrhea	Oral (MPNM).	[69]
			R	Gonorrhea	The root is chewed.	[49]
			L	Diarrhea	Fresh leaves are crushed, mixed with water, and given orally.	[71]
		Atuch (Am)	Wp	Diarrhea	Whole parts is pounded and mixed with drunk then drunk orally.	[44]
161. *Vernonia adoensis* (Sch. Bip. ex Walp.)H.Rob.	Asteraceae	Etse Mossie/Mererug (Am)	R	Diarrhea	Decoction.	[57]
162. *Vernonia amygdalina* Delile	Asteraceae	Eebicha (Or)	L, Sd	Diarrhea	Fresh leaf is pounded and seeds mixed butter and eaten orally.	[43]
			R	Diarrhea	Fresh root is crushed and mixed with honey.	[50]
		Grawa (Am)	—	Diarrhea	Crush or pound and mix with little water then drink for 5 days.	[59]
163. *Vernonia auriculifera* Hiern.	Asteraceae		R	Dysentery	Oral (MPNM).	[86]
164. *Withania somnifera* (L.) Dunal.	Solanaceae	Baala ajo (Or)	L	Diarrhea	Leaf is crushed and squeezed, and drunk orally.	[50]
		Gizawa (Am)	R	Gonorrhea, Syphilis	NM (MPNM).	[70]
		NM	L	Diarrhea	Leaf is crushed, squeezed, and mixed with water, and drunk orally.	[71]
		NM	R	Typhoid	Oral (MPNM).	[64]
		Egiziwa (Am)	L	Diarrhea	Crushing and oral.	[77]
165. *Ximenia americana* L.	Olacaceae	Enkoye (Am)	R	Gonorrhea	Crushed the root and filter then drink one cup for 3–5 days.	[87]
166. *Zingiber officinale* Roscoe	Zingiberaceae	Zingibil (Am)	Rh	Diarrhea	Chew and swallow the juice.	[68]
			Rh	Bone TB	Oral.	[69]
167. *Ziziphus mauritiana* Lam.	Rhamnaceae	Amurusam (Be)	Sd	Diarrhea	Seed is grind, dispersed in water and drunk.	[59]
168. *Ziziphus spina-christi* (L.) Desf	Rhamnaceae	Kurkura, Geba (Am)	R	Gonorrhea	NM.	[65]
			L	Diarrhea	NM.	[91]

Abbreviations: Af, Afar; Am, Amhara; Ama, Amaro; Ba, bark; Be, Berta; Bu, bulb; F, fruit; L, leaf; Gur, Gurage; La, latex; MPNM, method of preparation not mentioned; NM, not mentioned; Or, Oromo; R, root; Rb, root bark; Rh, rhizome; Sb, stem bark; Sd, SD; Sd, Sd; So, Somali; St, stem; Tig, Tigrai; TB, tuberculosis; Wp, whole part.

#### 3.2.4. Bacterial Disease List and Medicinal Plants Used to Treat the Disease

The results of the review findings showed that diarrhea, which is caused by *Escherichia coli* [35], was the first bacterial disease treated with many traditional medicinal plant species (89 species), followed by gonorrhea (caused by *Neisseria gonorrhoeae*), which was treated by 43 species of medicinal plants. According to [94], diarrhea is the foremost cause of mortality in the developing countries, and Ethiopia, as part of these nations, is reported to be highly affected by such severe diseases. The disease is usually reported to be caused by drinking contaminated water [95], food [96], and hygiene [97]. Gonorrhea, which was reported to be treated by the second highest number of medicinal plant species, was reported to be treated by the highest number of traditional medicinal plant species in other reports also [19]. This showed that these two disease types might have the highest occurrence in the country. The other significant point to be noted here is that the leaf of the traditional medicinal plants was the primary medicinal plant part to be used as sources of traditional remedies, followed by roots (Table 3). This might be for the sake of conserving the medicinal plant species, as collecting leaves as a primary source of remedy rather than other parts might not lead the medicinal species to the threat of loss, as explained in the work of [98]. The other might be because of ease of preparation and collection.

#### 3.2.5. Parts Used, Method of Preparation and Types of Antidotes Added

**Figure 2:**
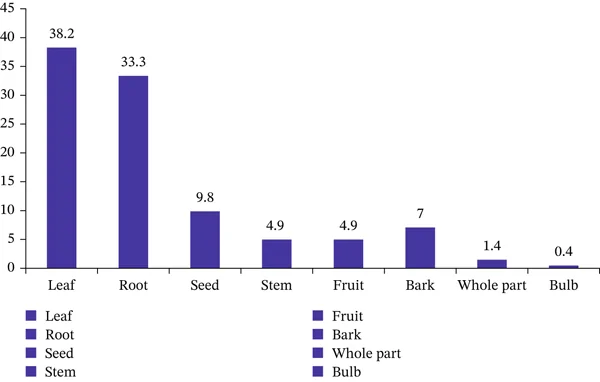
Proportion of the parts of medicinal plants based on their use for traditional medicine.

**Figure 3:**
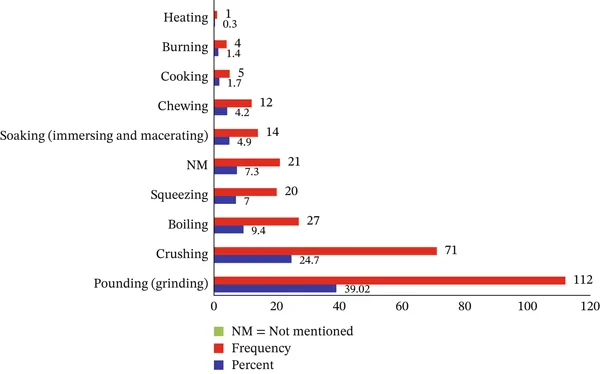
Proportion of the method of preparation.

The results of the current review findings showed that leaf (38.2%) was mentioned as the primary source of remedy for the preparation of traditional remedies (Figure 2). This might be because leaves were easier to prepare than the other parts. Additionally, these parts might mostly be found on the plants for a longer time than other parts, such as fruits and flowers, which are mostly seasonal in nature compared with the previous parts. The other reason might be for the conservation and sustainable utilization of the species, as utilizing roots, bark, bulbs, stems, and the whole parts is mostly a danger to the survival of the species rather than utilizing leaves. The results of the review showed that utilizing pounding as a preparation method of the traditional medicinal plant remedy was the most frequently used method than other types (Figure 3). This might be because most medicinal plant remedies were prepared after the parts were dried. Such types of preparation methods might lead to the loss of essential volatile chemicals from the medicinal plant species.

Regarding the materials added to traditional medicine or remedies, several types of substances were reported to be added to the primary sources of medicinal plants during the preparation process. However, some traditional medicines were reported to be prepared without the need for water, whereas other traditional medicine preparations need the use of water [45, 48, 50, 59–63]. Some others need the addition of Tella (in Amharic, local beer in Ethiopia) [44, 58], Areqie katicala (local liquor) [58], milk [44, 68], salt [49, 71, 73], Vaseline [82], yogurt [44, 58, 62]. Some others need the addition of butter [43, 49, 53, 55]. Moreover, the review of the findings showed that different traditional medicines were prepared with the addition of sugar [49, 53, 73]. Cheese [70] and oil [87] were also reported as the additional antidotes of traditional medicine, as the review of the findings shows. Furthermore, honey is the other additional antidote for the preparation of the traditional remedy, as in most findings reported [43–45, 49, 50, 53, 54, 57, 58, 68, 71, 73, 75, 78, 81, 82, 84, 85]. Tea [44] and honey coffee or coffee sugar [78, 87] were also reported as one of many types of antidotes used with medicinal plants for the preparation of traditional remedies.

In some reports, plants themselves were reported to be used as antidotes. The powder, juices and other parts of plants were reported to be used as sources of antidotes for the preparation of traditional remedies (Table 4). Dried or fresh bulbs of *Allium cepa* [53, 72], the seed of *Nigella sativa* and the rhizome of *Zingiber officinale* (coffee) [58, 72] were reported to be used as antidotes. Coffee [58, 61, 75, 80, 82, 88], leaves of *Croton macrostachyus* [47] were the other reported antidotes. Moreover, leaves of *Cuminum cyminum* and *Tragia cinerea* [61], the roots of *Gnidia glauca* [53], seeds of *Lepidium sativum* L. and *sorghum* flour [54], *Fagaropsis angolensis*, *Acmella caulirhiza,* and the internal part of the stem bark of *C. macrostachyus* were used as antidotes [81]. *Cucumis ficifolius* fruit [74] was one of the antidotes. Garlic [46] was one of the common antidotes.

The roots of *Solanum incanum*, *Osyris quadripartita* [81], and *C. aurea* [53] were also used as antidotes. To mention, some other reported additional antidotes of the traditional remedy, the leaves of *C. aurea* [48], *Clematis simensis* [53], *F. angolensis*, *A. caulirhiza*, *Clematis hirsuta*, *Ehretia obtusifolia*, *C. macrostachyus,* and *Teclea simplicifolia* [81], and *Impatiens rothii* [54] were the other reported antidotes. Lemon liquid [70], *Ximenia americana* seed oil [83], powder of wheat or barley [62], *Eragrostis tef* [87] were mentioned to be used as antidotes. Qocho (*Ensete ventricosum* bread) especially in the southern parts of Ethiopia was reported to be used as antidotes, and stem bark of *C. macrostachyus* [81] was the other source of antidote.

#### 3.2.6. Route of Administration and Method of Application

**Figure 4:**
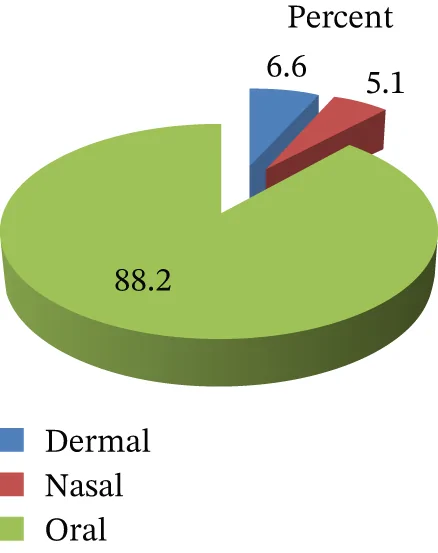
Types of the routes of administration.

**Figure 5:**
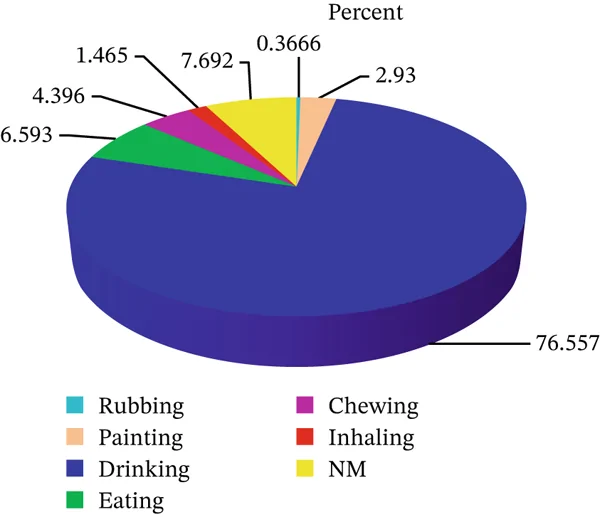
Different methods of application; NM, not mentioned.

The result of the review work showed that the local communities in different parts of Ethiopia utilized different types of administration after preparing the traditional remedy. Accordingly, three types of routes were reported, and most remedies were administered orally (Figure 4). This demonstrated that the sources of the parasites′ infections were predominantly internal rather than dermal or optical, which need other methods of administration. Like the routes of administration, there were reported several methods of application for the prepared remedy. Out of these, drinking took the first rank (Figure 5). This indicated that most of the remedy was prepared in the form of a solution or liquid form which was suitable for drinking.

### 3.3. Antibacterial Activities of Medicinal Plants and Determinant Factors

Different experimental reports enumerated that bacterial growth inhibition varied because of different factors such as bacterial species type, type of solvent, concentration difference of the plant extracts, the parts, and the type of the medicinal plant (Table 5).

**Table 5:** Bacterial species, types, medicinal plant, parts used, MIC (milligram/milliliter), inhibition zone (in millimeter) of bacterial growth, and citation.

No	Bacterial species	Type	Medicinal plant species				Citation
			Species	PU	MIC (mg/mL)	Inhibition zone (mm)	
1.1.	*Bacillus pumilus*	Gram‐positive	*Aloe trigonantha*	La	200	6.0	[99]
1.2.	*Bacillus subtilis*	Gram‐positive	*A*. *trigonantha*	La	200	6.0	[99]
2.	*Citrobacter freundii*	Gram‐negative	*Acokanthera schimperi*	L	25	7.67	[100]
3.1.	*Enterococcus faecalis*	Gram‐positive	*Achyranthes aspera*	L	4.0	NG	[101]
			*Embelia schimperi*	S/F	512	NG	[66]
			*Ocimum gratissimum*	L	512	NG	
			*Rubus steudneri*	R	512	NG	
			*Rumex nepalensis*	R	512	NG	
4.1.	*Escherichia coli*	Gram‐negative	*Aloe vera*	L	100	12.6	[102]
			*Allium sativum*	Bu	10	13	[103]
			Chloramphenicol	PC	10	25	[103]
			*Calpurnia aurea*	L	50	2.66	[104]
			*Cordia africana*	L	100	11.6	[101]
			*Croton macrostachyus*	L	50	1.8	[104]
			*Erythrina brucei*	L	50	0.0	[104]
			*Eucalyptus globulus*	L	100	16.5	[101]
			*Jasminum abyssinicum*	L	25	NA	[105]
			*Kosteletzkya begonifolia*	L	50	21.67	[106]
			*Lagenaria siceraria*	F/Sd	25	NA	[105]
			*Leucas martinicensis*	L	50	20.33	[106]
			*Nicotiana tabacum*	L	50	1.7	[104]
			*Ranunculus multifidus*	L	50	21.67	[106]
			*Solanecio gigas*	L	25	NA	[105]
			*Verbena officinalis*	L	100	11.8	[101]
			*Vinca major*	L	100	6.00	[102]
			*Withania somnifera*	R	50	1.7	[104]
			*Zingiber officinale*	R	100	9.7	[102]
			Streptomycin	PC	50	4.9	[104]
5.1.	*Klebsiella pneumoniae*	Gram‐negative	*A*. *aspera*	L	5.3	NG	[117]
			*A*. *schimperi*	L	25	8.67	[100]
6.1.	*Proteus mirabilis*	Gram‐negative	*A*. *aspera*	L	32.0	NG	[101]
7.1.	*Pseudomonas aeruginosa*	Gram‐negative	*J. abyssinicum*	L	25	18.00	[105]
			*C. aurea*	L	50	2.8	[104]
			*C*. *macrostachyus*	L	50	2.33	[104]
			*E*. *brucei*	L	50	0.0	[104]
			*K*. *begonifolia*	L	50	19.67	[106]
			*L*. *siceraria*	F	14.33	14.33	[105]
				Sd	25	16.00	[105]
			*L*. *martinicensis*	L	50	21.67	[106]
			Amoxicillin	PC	0.1	NA	[105]
			Chloramphenicol	PC	0.1	25.33	[105]
			*N*. *tabacum*	L	50	1.6	[104]
			*R*. *multifidus*	L	50	20.67	[106]
			*S*. *gigas*	L	25	14.67	[105]
			*W*. *somnifera*	R	50	1.6	[104]
			Streptomycin	PC	50	4.9	[104]
8.1.	*Salmonella Typhimurium*	Gram‐negative	*A. aspera*	L	32.0		[99]
			*A*. *vera*	L	100	9.13	[102]
			*C*. *africana*	L	100	9.1	[101]
			*E*. *globulus*	L	100	11	
			*K*. *begonifolia*	L	50	17.00	[106]
			*L*. *martinicensis*	L	50	19.33	[106]
			*R*. *multifidus*	L	50	20.33	[106]
			*V*. *major*	L	100	8.3	[102]
			*Z*. *officinale*	R	100	8.3	[102]
8.2	*Salmonela typhi*		*A*. *schimperi*	L	25	7.67 ± 0.33	[100]
9.1.	*Shigella flexneri*	Gram‐negative	*A*. *aspera*	L	13.3	NG	[101]
9.2.	*Shigella sonnei*	Gram‐negative	*Brucea antidysenterica*	L	100	5.5	[101]
			*C*. *africana*	L	100	8	
			*Otostegia integrifolia*	L	100	9.3	
			*V*. *officinalis*	L	100	13.1	
			*W*. *somnifera*	L	100	9.8	
10.1.	*Staphylococcus aureus*	Gram‐negative	*A*. *aspera*	L	4.0		[101]
			*A*. *vera*	L	100	9.0	[102]
			*B*. *antidysenterica*	L	100	7	[103]
			*C*. *aurea*	L	50	2.53	[104]
			*C*. *africana*	L	100	7.3	[101]
			*C*. *macrostachyus*	L	50	2.0	[104]
			*E*. *brucei*	L	50	0.0	[104]
			*E*. *globulus*	L	100	13	[101]
			*A*. *sativum*	Bu	10	16	[103]
			Chloramphenicol	PC	10	32	[103]
			*J. abyssinicum*	L	25	18.00	[105]
			*K*. *begonifolia*	L	50	17.00	[106]
			*L*. *siceraria*	F	25	13.67	[105]
				Sd	25	14.0	[105]
			*L*. *martinicensis*	L	50	19.00	[106]
			*N*. *tabacum*	L	50	1.8	[104]
			*O*. *integrifolia*	L	100	9.1	[103]
			*R*. *multifidus*	L	50	20.67	[106]
			*S*. *gigas*	L	25	16.0	[105]
			Amoxicillin	PC	0.1	29.33	[105]
			Chloramphenicol	PC	0.1	40.0	[105]
			Streptomycin	PC	50	4.9	[104]
			*V*. *officinalis*	L	100	9	[101]
			*V*. *major*	L	100	0.00	[102]
			*W*. *somnifera*	L	100	8.8	[103]
				R	50	1.7	[104]
			*Z*. *officinale*	R	100	8.8	[102]
10.2.	*Staphylococcus epidermidis*	Gram‐positive	*A*. *aspera*	L	10.7	NG	[101]
11.1.	*Streptococcus agalactiae*	Gram‐positive	*A*. *aspera*	L	5.3	NG	[101]
11.2.	*Streptococcus pyogenes*	Gram‐negative	*C*. *macrostachyus*	L	256	NG	[66]
			*Dodonaea angustifolia*	L	512	NG	
			*E*. *schimperi*	S/F	128	NG	
			*J. abyssinicum*	L	25	18.00	[105]
			*L*. *siceraria*	F	25	13.33	[105]
				Sd	25	14.67	[105]
			Amoxicillin	PC	0.1	30.67	[105]
			Chloramphenicol	PC	0.1	40.00	[105]
			*Maesa lanceolata*	L	256	NG	[66]
			*O*. *gratissimum*	L	128	NG	
			*Olinia rochetiana*	L	512	NG	
			*R*. *steudneri*	R	512	NG	

Abbreviations: Bu, bulb; F, fruit; F/S, fruit/stem; L, leaf; La, latex; MIC, microorganisms/minimum inhibitory concentration; NA or sign “—,”not active/no activity; NC, negative control; NG, not given; PC, positive control; PU, parts used; S, stem; SB, stem bark; Sd, seed.

#### 3.3.1. Types of Extraction Solvents and Antibacterial Activity

The different antibacterial test studies assured that the difference in the type of extraction solvents alters the antibacterial activity of the extracted materials of medicinal plants. The antibacterial activity of the leaf extracts of *Leucas martinicensis* using petroleum against *P*. *aeruginosa* (standard bacteria) was 23.67 ± 3.67, but its chloroform and methanol extracts were 22.00 ± 4.50 (the same result) [106] which was lower than the previous. The minimum inhibitory concentration of the Dichloromethane solvent leaf extract *of Acokanthera schimperi* against *Klebsiella pneumoniae* was 4.17 ± 0.00 but its petroleum ether solvent extract was 11.13 ± 2.78, which showed that the antibacterial activity varies because of its extraction solvent [100]. Other findings showed that the antibacterial activity of *Dodonea angustifolia*, *O. quadripartita* and *Plantago lanceolata* extracts showed that there was greater variation in antibacterial activity based on solvent type, and it demonstrated that the antibacterial activity of ethanol extracts of the species was higher than that of other solvent (hexane, acetone) extracts against *Listeria monocytogenes* and *S*. *aureus* with inhibition zones of 14.5 mm [107]. Similarly, n‐hexane and methanolic leaf extracts of *Myrtus communis* showed maximum antibacterial activity against *E. coli* and *S*. *aureus* strain with inhibition zones of 5.67–5.5 mm and the least activity was observed in chloroform and methanol extracts with inhibition zones of 1–2.2 mm against *S*. *aureus* [108].

According to [109], n‐hexane and methanolic leaf extracts of *M. communis* showed maximum antibacterial activity against *E. coli* and *S*. *aureus* strain with inhibition zone of 5.67–5.5 mm and the least activity was observed in chloroform and methanol extract with inhibition zone of 1–2.2 mm against *S*. *aureus* [108]. Strong inhibitions were recorded for methanol and chloroform extracts of *L. martinicensis* and *Ranunculus multifidus* against *E. coli* (26.67 ± 3.33) and *S*. *aureus* (26.67 ± 3.33 mm) at 200‐mg/mL concentration, respectively. Relatively, similar activity was observed even at 100‐mg/mL concentration of petroleum ether extract of *L. martinicensis* against *P. aeruginosa* (24.00 ± 3.50) [106]. Furthermore, additional studies illustrated that chloroform leaf extract of *Kosteletzkya begonifolia* showed the least activity against *Salmonella Typhimurium* (14.33 ± 2.33) at both 50 and 30 *μ*g/mL [106]. It further illustrated that ethanol extracts of leaves (25 mg/mL) of *Acanthus sennii* showed high antibacterial activity against *S*. *aureus* (standard strain) with an inhibition zone of 14 ± 0.6 mm. Ethanol extracts of buds (100 mg/mL) of this plant species further showed high antibacterial activity against the same species but at different concentrations [110]. The solvent leaf extracts of *Lantana camara* showed different degrees of antibacterial activity against different bacterial strains [111]. *S*. *aureus* was more susceptible to the petroleum solvent leaf extracts of this plant species than chloroform extracts with an inhibition zone of 14.17 ± 0.29 mm to 17.7 ± 0.2 mm. However, *P*. *aeruginosa* was reported to be less susceptible to these solvent extracts, with an inhibition zone of 7.9 ± 0.21 mm to 11.3 ± 0.5 mm. Acetone root extract of the other plant species, *Verbascum sinaiticum*, showed the highest in vitro antibacterial activity against *Bacillus subtilis* (7–8 mm) (gram‐positive bacteria) and *Klebsiella aerogenes* (7–10 mm) (gram‐negative bacteria), *E. coli* (8–10 mm), and *Vibrio cholerae* (7–10 mm), whereas ethanol root extract of the species showed less in vitro antibacterial activity against *E. coli* (gram‐negative bacteria) (7–11 mm) [112]. These results showed that the antibacterial activity of the medicinal herb has been determined not only by the type of the solvent, but it has also been dominantly determined by the types of the medicinal plant species and the type of the bacterium itself.

#### 3.3.2. Plant Species and Antibacterial Activity

The results showed that the minimum inhibitory concentration of medicinal plants varies from species to species (Table 5). As literature sources implicated that some species of plants had a minimum growth inhibition concentration, whereas some others with a better amount of inhibition concentration, and some others completely with no growth inhibition. As the findings of [104] illustrated that the methanol and chloroform extracts of *Erythrina brucei* showed no growth inhibition against *S. aureus, E. coli*, and *P. aeruginosa*. To add more, *L. sativum* did not exhibit an inhibitory property against *Bacillus cereus* [113]. Furthermore, the extracts of *C. aurea*, *Carissa spinarum*, *C. hirsuta*, *Clarkia cylindrica*, *Thalictrum rhynchocarpum*, and *V. sinaiticum* were also reported to have no antibacterial activity against some tested bacterial species: *B. cereus*, *Clostridium perfringens*, *L. monocytogenes*, *Staphylococcus epidermidis*, *Enterococcus faecalis*, *S*. *aureus*, *Streptococcus pyogenes*, *Bacteroides fragilis*, *E. coli*, *P*. *aeruginosa*, *Salmonella enteritidis* [66]. The experimental studies of [114] further elaborated that the extract of *Polysphaeria aethiopica* was reported to have zero inhibition zone against *E. coli*. The extracts of *Vinca major* showed the least antibacterial activity against *S. Typhimurium*, *E. coli*, *S. aureus,* and *Streptococcus agalactiae* than other test plant species [102].

On the contrary, the extract of many medicinal herbs was reported with effective bacterial growth inhibition zone. Among which, the extracts of *Euphorbia depauperata*, *Rumex abyssinicus*, and *Lippia adoensis* showed the highest antibacterial activity against *E. coli* [114]. Additional studies further presented that the extract of *Aloe vera* showed high antibacterial activity against *E. coli* with inhibition zone of 18.2 ± 0.91 mm and *S. aureus* with inhibition zone of 14.5 ± 0.48 mm. The experimental results of [115] also assured that the extracts of *Pterolobium stellatum* were found to be the most active against *Salmonella typhi*, *Salmonella paratyphi*, *P. aeruginosa*, *S. aureus*, and *E. coli*. The leaf and twig extracts of *Embelia schimperi* were also reported to have the strongest antibacterial activity against *B. cereus*, *L. monocytogenes*, and *S. pyogenes* [66]. Some plant extracts were reported to have even higher antimicrobial activity than standard common drugs such as tetracycline, chloramphenicol, and penicillin. As evidence for this, the experimental results of [103] reported that the extracts of *Allium sativum* (25 mg/mL) showed a higher growth inhibition zone (28 and 26 mm, respectively) against *S. aureus* and *E. coli* than Tetracycline (30 mg/mL) which was reported to have 22‐ and 16‐mm inhibition zone against these bacterial species, respectively.

#### 3.3.3. Bacteria Type and Antibacterial Activity

The results of the present study showed that the antimicrobial activity of medicinal plant extracts varies from one pathogen to the other. The experimental results of [103, 115] showed that the antimicrobial activity on standard bacteria is greater than the clinical isolates. The strongest antibacterial activity (64 mg/mL) was shown by leaf and twig extracts of *E. schimperi* against *B. cereus*, *L. monocytogenes*, and *S. pyogenes*. Moreover, seed and fruit extracts of this species also showed strong activity (MIC of 128 mg/mL) against *B. cereus* and *S. pyogenes*. Correspondingly, strong growth inhibitory activities (MIC of 128 mg/mL) were observed for extracts of *Ocimum gratissimum* against *S. pyogenes*, and those of *Rubus steudneri* against *S. epidermidis*. Extracts of *R. steudneri* were the only extracts to show antimicrobial activity against *P. aeruguinosa* and *S. enteritidis* (gram‐negative bacteria). Two of the gram‐negative bacteria, that is, *B. fragilis* and *E. coli*, were resistant to all extracts tested in this study. Among the gram‐positive bacteria, *C. perfringens* was found to be less sensitive as inhibited by the extracts of only two species, *E. schimperi* and *O. gratissimum*. It was reported that gram‐positive bacteria were generally found more susceptible to the extracts than the gram‐negative ones [66]. Other experimental studies further agreed that the extracts of *Agave americana* were reported to show more effective antibacterial activity against gram‐positive bacteria than their gram‐negative complements [26]. The growth of different bacterial species is further determined by the concentration of different extract materials. It was reported that the antimicrobial activity of the leaf extracts (50 mg/mL) of *R. multifidus* against *S. aureus* was 20.67 ± 1.17 mm but at the concentration of 200 mg/mL it was 22.00 ± 0.50 mm [106].

As some additional experimental results further illustrated that *S. aureus* and *E. coli* (standard bacterial strains) were completely inhibited by 10‐ and 15‐mg/mL of agar media, respectively, and their clinical isolates were completely inhibited by 25 mg/mL, indicating that standard isolates were the most sensitive and clinical isolates were the least sensitive ones [103]. It was also reported that most plant extracts accomplished smaller zones of inhibition against test bacteria than reference bacterial species due to the probability of antibacterial resistance in isolate species [116]. Other results showed that the leaf extracts of *L. camara* against *E. coli* and *S*. *aureus* showed more inhibition than *P*. *aeruginosa* [111]. *S*. *aureus* was susceptible to the methanol extract of seeds (1.25 mg/mL) and ethyl acetate extract of root barks (2.5 mg/mL) of *Moringa stenopetala* with inhibition zones of 18.66 ± 0.88 mm and 16.00 ± 1.15 mm, respectively, but *P*. *aeruginosa* was the most resistant bacteria to all extracts [117].

Besides, the ethanol extracts of the bud of *A. sennii* against *S*. *aureus* (standard strains) showed the highest antibacterial activity and its chloroform extract had no antibacterial activity against standard strains and clinical isolates of *E. coli* [110]. The results showed that there was a difference in the antibacterial activity of medicinal plant extracts because of whether the bacteria were isolated in laboratory or standard bacteria [116]. In contrast, the methanol extracts of *Solanecio gigas* showed better antibacterial activity against *S. aureus* (standard strain) as compared with *S. aureus* (clinical isolate) at 100‐ and 50‐mg/mL concentrations, and equal activity at 25 mg/mL [105]. Other experimental data of the present article showed that *Rumex nepalensis* showed the highest inhibition against *Salmonella* species and no inhibition against *P. aeruginosa* [116]. This might be related to the type of the bacteria treated with the extracted materials.

#### 3.3.4. Variation in Minimum Inhibitory Concentration (MIC) Across Bacterial Species

**Figure 6:**
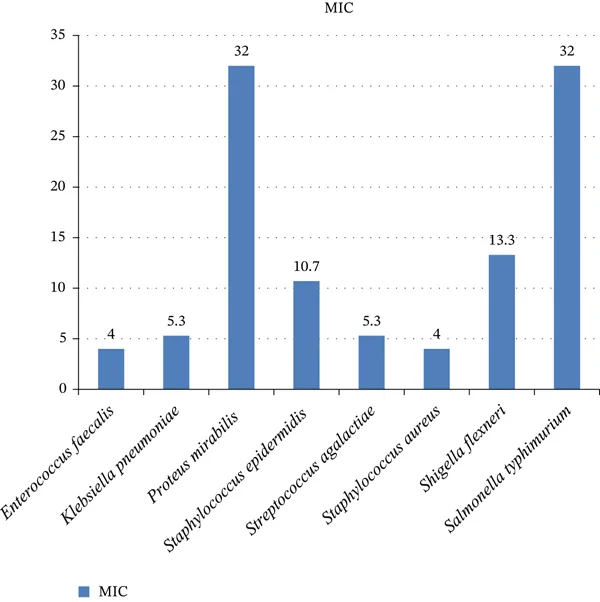
MIC difference across different bacterial species.

The findings of the current article showed that *E. faecalis* and *S. aureus* were reported to be inhibited at the lowest MIC whereas the highest concentration of the plant extracts was needed for the inhibition of *Proteus mirabilis* and *S. Typhimurium* growth (Figure 6). The MIC of *E. schimperi* against *B. cereus*, *C. perfringens*, *E. faecalis*, and *L. monocytogenes* was 128 mg/mL, 512 mg/mL, 512 mg/mL, and 256 *μ*g/mL, respectively, showing the antibacterial activity is determined by the concentration of plant extracts. As illustrated by an appreciable number of research sources, the highest concentration of the plant extracts is needed especially for increasing the bactericidal effects of the extracts, meaning that the lowest extraction concentration was reported to be the lowest in its antibacterial activity, and in its bactericidal activity; on the other hand the reverse is true according to several experimental findings. Thus, concentration is the other additional limiting factor of the plant extracts for playing their active role in antibacterial activity (Table 5).

### 3.4. Phytochemical Analysis

**Figure 7:**
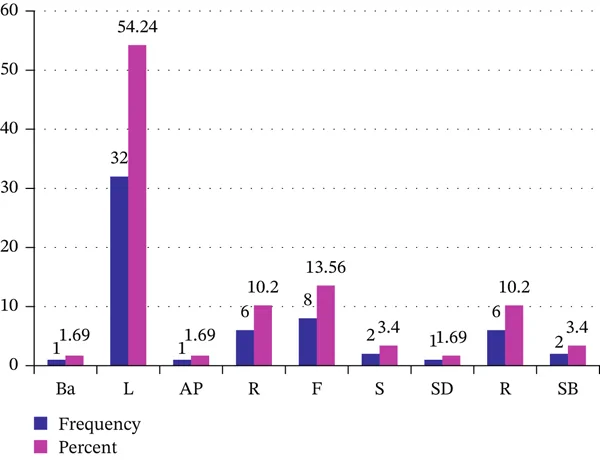
Proportion of plant parts presented for phytochemical analysis.

The results of the present findings showed that seven major types of phytochemicals, namely phenol, flavonoid, tannin, terpenoid, phytosterol, alkaloid, and glycoside were identified in the different plant parts including leaf, fruit, stem, stem bark, seed and some other aerial parts of the species (Table 6) even if the majority of the studies (54.24%) were undergone on the leaves of plants (Figure 7). Tannin was reported to be found with the highest percentage of frequency in most of the studied plant parts, followed by saponin, and all the tested phytochemicals were reported to be detected in the leaves. However, this might be related to the proportion of the plant parts presented for test (Table 7). However, some experimental results illustrated that some plant parts were reported as lacking some of these phytochemicals. As evidence for this, Saponin was not noticed in the tested roots of *Asparagus africanus* and *Stephania abyssinica*, and in the leaves of *Hypericum revolutum* and *Otostegia integrifolia*. Alkaloids, flavonoids, saponins, and terpenoids were not observed in the fruits of *Balanites aegyptiaca*. The tested leaves of *Brucea antidysenterica* lacked phytosterol and glycosides. The tested leaves of *C. spinarum* lacked phytosterols.

**Table 6:** Different phytochemicals found in medicinal plants and citation.

Species	PU	Phytochemicals								Citation
		Sa	Ph	Fl	Ta	Ter	Pht	Al	Gly	
1. *Achyranthes aspera*	L	+	+	+	+	−	+	+	NT	[101]
2. *Acokanthera schimperi*	L	+	+	+	+	+	NT	+	−	[69]
3. *Ajuga integrifolia*	AP	+	+	+	NT	+	+	+	+	[118]
4. *Artemisia afra*	L	+	+	+	NT	+	+	+	+	[118]
5. *Artemisia absinthium*	L	+	+	+	NT	+	+	+	+	[118]
6. *Asparagus africanus*	R	−	+	+	+	+	+	+	NT	[119]
7. *Balanites aegyptiaca*	F	−	+	−	NT	−	NT	−	NT	[25]
8. *Bersama abyssinica*	S	+	+	+	+	+	+	NT	+	[120]
9. *Brucea antidysenterica*	L	+	+	+	+	+	−	+	−	[121]
10. *Calpurnia aurea*	L	+	+	+	+	+	+	+	+	[65]
11*. Carissa spinarum*	L	+	+	+	+	+	−	+	+	[122]
12. *Cirsium englerianum*	F	+	+	+	+	+	NT	+	+	[114]
13. *Cordia africana*	L	+	+	+	+	NT	NT	+	+	[123]
14. *Croton macrostachyus*	SB	+	+	+	+	+	+	+	+	[124]
15. *Cucumis pustulatus*	R	+	+	+	+	NT	NT	−	+	[114]
16. *Discopodium penninervium*	L	+	−	+	+	NT	NT	+	+	[114]
17. *Dodonaea angustifolia*	L	+	+	+	+	+	+	+	+	[124]
18. *Eucalyptus globulus*	L	+	+	+	+	NT	NT	+	+	[123]
19. *Euphorbia depauperata*	Ba	+	+	+	−	+	NT	+	+	[114]
20. *Ferula communis*	R	+	+	+	+	+	+	+	NT	[119]
21. *Foeniculum vulgare*	L	+	+	+	+	NT	NT	+	+	[123]
22. *Gnidia involucrata*	F/S	+	+	+	+	+	+	−	NT	[121]
23. *Grewia ferruginea*	SB	+	NT	+	+	+	NT	NT	NT	[116]
	L	+	NT	+	+	+	NT	NT	NT	[116]
24. *Hagenia abyssinica*	F	+	+	+	+	+	+	−	+	[125]
25. *Hypericum revolutum*	L	−	NT	+	+	+	NT	−	NT	[116]
26. *Justicia schimperiana*	L	+	+	+	+	−	+	+	NT	[126]
27. *Lantana camara*	L	+	+	+	+	+	NT	+	+	[111]
28. *Lippia adoensis*	L	+	+	+	+	+	NT	+	−	[114]
29. *Ocimum gratissimum*	L	+	+	+	+	+	+	−	−	[127]
30. *Osyris quadripartita*	L	+	+	+	+	+	−	+	−	[107]
31. *Otostegia integrifolia*	L	−	+	−	NT	−	NT	−	NT	[25]
32. *Plantago lanceolata*	L	+	+	+	+	+	−	+	−	[107]
33. *Phytolacca dodecandra*	L	+	+	+	+	NT	NT	+	+	[123]
	F	+	NT	+	+	−	NT	NT	NT	[116]
34. *Polysphaeria aethiopica*	L	+	+	+	+	+	+	+	−	[114]
35. *Psidium guajava*	L	+	+	−	NT	−	+	−	−	[128]
36. *Pterolobium stelatum*	R	+	NT	−	+	+	NT	−	NT	[116]
37. *Rhamnus prinoides*	L	+	+	+	+	−	−	+	NT	[126]
38. *Rumex abyssinicus*	R	+	+	+	+	+	+	+	+	[114]
39. *Rumex nepalensis*	L	+	NT	+	+	−	NT	NT	NT	[116]
40. *Ruta chalepensis*	F	+	+	+	+	+	−	+	+	[127]
41. *Solanum incanum*	F	+	+	+	+	+	−	−	+	[127]
42. *Solanum sisymbriifolium*	L	+	+	+	−	+	+	+	NT	[129]
43. *Solanum somalense*	F	+	−	+	−	+	+	+	−	[65]
44. *Stephania abyssinica*	R	−	+	−	+	−	−	+	NT	[119]
45. *Tamarindus indica*	Sd	+	−	−	NT	+	NT	−	NT	[25]
46. *Thymus schimperi*	L	+	+	+	+	−	−	+	NT	[125]
47. *Verbascum sinaiticum*	L	+	+	+	+	+	+	+	+	[65]
48. *Verbena officinalis*	L	+	+	+	+	NT	NT	+	+	[101]
49. *Vernonia amygdalina*	L	+	+	+	+	+	+	+	NT	[119]
50. *Withania somnifera*	L	+	+	+	+	NT	NT	+	+	[130]

Abbreviations: Al, alkaloid; AP, aerial part; F, fruit; F/S, fruit/stem; Fl, flavonoid; Gly, glycoside; L, leaf; NT, no test (test not conducted for this component); Ph, phenol; PHt, PHytosterol; PU, parts used; S, stem; Sa, saponin; SB, stem bark; Sd, seed; Ta, tannin; Ter, terpenoid.

**Table 7:** List of phytochemicals and frequency of the phytochemicals per plant parts tested.

Phytochemicals	Present (+), absent (−)	Frequency per parts tested							Total frequency	Percent	Rank
		AP	Ba	F	L	R	S	SD			
Saponin	+	1	3	6	30 (63.8%) ^∗^	4	2	1	47	88.7	2nd
	−	0	0	2	2	2	0	0	6		
	NT	0	0	0	0	0	0	0	0		

Phenol	+	1	3	6	31 (62%) ^∗^	6	2	1	50	86.2	4th
	−	0	0	1	1	0	0	1	3		
	NT	0	1	0	3	1	0	0	5		

Flavonoid	+	1	1	7	30 (66.7%) ^∗^	4	2	0	45	88.2	3rd
	−	0	0	1	2	2	0	1	6		
	NT	0	0	0	0	0	0	0	0		

Tannin	+	0	0	6	29 (67.4%) ^∗^	6	2	0	43	93.5	1st
	−	0	1	1	1	0	0	0	3		
	NT	1	0	1	4	0	0	1	7		

Terpenoid	+	1	1	5	18 (56.3%) ^∗^	4	2	1	32	76.2	5th
	−	0	0	2	7	1	0	0	10		
	NT	0	0	0	7	1	0	0	8		

Phytosterol	+	1	0	2	12 (60%) ^∗^	3	2	0	20	69	7th
	−	0	0	2	6	1	0	0	9		
	NT	0	1	3	12	2	0	1	19		

Alkaloid	+	1	1	3	26 (74.3%) ^∗^	4	0	0	35	74.5	6th
	−	0	0	4	4	2	1	1	12		
	NT	0	0	1	2	0	1	0	4		

Glycoside	+	1	1	4	14 (58.3%) ^∗^	2	1	1	24	68.6	8th
	−	0	0	1	8	0	2	0	11		
	NT	0	0	3	10	4	1	1	19		

*Note:*  ^∗^: Dominantly tests were conducted on leaves than on other parts.

Abbreviations: AP, aerial part; F, fruit; F/S, fruit/stem; L, leaf; NT, no test (test not conducted for this component); PU, parts used; S, stem; SB, stem bark; Sd, seed.

In contrast, the leaves of *C. aurea*, and the roots of *R. abyssinicus* possessed all the seven phytochemicals and some other tested plant parts also lacked any one of the mentioned phytochemicals and so on (Table 6). This further showed that some parts of plants might be enriched with many phytochemicals; some others might possess some of the phytochemicals, whereas some others might also be free from such essential chemicals. However, as many research findings indicate that phytochemicals that are not present in different plant parts of several species in one test might be found in that same plant part of the same species in another test because of different reasons such as the extraction method and procedure technique difference or difference in the extraction solvent of the phytochemicals might be one of such reasons as the results of different research findings demonstrated [25, 114]. This might be as a result of one solvent having the ability to extract different phytochemicals in an efficient manner, whereas some others might fail.

Thus, the function of the extracted materials from the various medicinal plant parts might be related to the type of plant species which possess or lack the essential phytochemicals, and the types of solvents which determine the amount and the types of phytochemical found in the extracted materials used for the test. That is why, as demonstrated in the above section of the discussion, some extracted plant materials were reported with no activity on the experimental bacterial species, some others with moderate activity, and some others also with the most effective antimicrobial activity. Therefore, the pharmaceutical effectiveness of the extracted materials of medicinal plant species might be related to not only such crucial phytochemicals, but also the effectiveness of solvents applied for extraction. The difference in the type of extraction solvent brings about a difference in antibacterial activity. The antimicrobial activity of the leaf extracts of *L. martinicensis* using petroleum against *P. aeruginosa* (standard bacteria) was 23.67 ± 3.67, but its chloroform and methanol extracts had an inhibition of 22.00 ± 4.50 mm, which was lower than the previous solvent extraction [106]. In addition, its water (aqueous) extracts showed zero activity on *E. coli* and *S. aureus* [110, 131].

## 4. Conclusion

Bacterial diseases were reported to be the major causes of illness and mortality in Ethiopia. These diseases have become a great burden to the socioeconomy of the country. Apart from the cost of antibiotics used for the treatment of the diseases, the resistance ability of these diseases to different modern antibiotics has become a major threat in Ethiopia. Because of such and other pushing factors, the great proportion of the country′s population depends on traditional medicines derived from medicinal plants. Currently, ethnobotanical studies show that many species of medicinal plants (168 species) were reported to treat the 10 recognized bacterial pathogens. Reports further showed that diarrhea had the highest prevalence and was treated with the greatest number of medicinal plants (89 species). In addition, the different phytochemical studies showed that plant species were the reservoirs of multiple types of metabolites which might enable the medicinal species in fighting pathogenic bacteria. Different antibacterial test studies further illustrated that plant‐derived extracts showed an ability to inhibit the growth of different bacterial species and also had a killing ability of the pathogen. As the concluding idea, the medicinal plant has the power of treating bacterial pathogens. The power of medicinal plants was because of possessing different essential phytochemicals. Moreover, organized and strengthened ethnobotanical exploration should be conducted in parallel to the continued and strengthened phytochemical and antibacterial studies. In general, medicinal plants might be the future hope for drug discovery for the headache and burden bacterial diseases if efforts for conservation of medicinal species and drug discovery of the species are continued sustainably.

## Author Contributions

The author (Derebe Alemneh) is the sole responsible person for all of the works reported including data gathering, data analysis and synthesis, interpretation, drafting, revision, and final writing of the manuscript.

## Funding

No funding was received for this manuscript.

## Consent

The author has nothing to report.

## Conflicts of Interest

The author declares no conflicts of interest.

## Data Availability

All the data sources extracted from the gathered articles were incorporated in the manuscript. Thus, the author has nothing to report.
